# Selection of reference genes for quantitative real-time PCR in equine *in vivo *and fresh and frozen-thawed *in vitro *blastocysts

**DOI:** 10.1186/1756-0500-2-246

**Published:** 2009-12-11

**Authors:** Katrien Smits, Karen Goossens, Ann Van Soom, Jan Govaere, Maarten Hoogewijs, Emilie Vanhaesebrouck, Cesare Galli, Silvia Colleoni, Jo Vandesompele, Luc Peelman

**Affiliations:** 1 Department of Reproduction, Obstetrics and Herd Health, Faculty of Veterinary Medicine, Ghent University, Salisburylaan 133, B-9820 Merelbeke, Belgium; 2 Department of Nutrition, Genetics and Ethology, Faculty of Veterinary Medicine, Ghent University, Heidestraat 19, 9820 Merelbeke, Belgium; 3 Laboratorio di Tecnologie della Riproduzione, AVANTEA srl, Cremona, Italy; 4 Center for Medical Genetics, Ghent University Hospital, De Pintelaan 185, 9000 Gent, Belgium; 5 Dipartimento Clinico Veterinario, Università di Bologna, Italy

## Abstract

**Background:**

Application of reverse transcription quantitative real-time polymerase chain reaction is very well suited to reveal differences in gene expression between *in vivo *and *in vitro *produced embryos. Ultimately, this may lead to optimized equine assisted reproductive techniques. However, for a correct interpretation of the real-time PCR results, all data must be normalized, which is most reliably achieved by calculating the geometric mean of the most stable reference genes. In this study a set of reliable reference genes was identified for equine *in vivo *and fresh and frozen-thawed *in vitro *embryos.

**Findings:**

The expression stability of 8 candidate reference genes (*ACTB*, *GAPDH*, *H2A/I*, *HPRT1*, *RPL32*, *SDHA*, *TUBA4A*, *UBC*) was determined in 3 populations of equine blastocysts (fresh *in vivo*, fresh and frozen-thawed *in vitro *embryos). Application of geNorm indicated *UBC*, *GAPDH*, *ACTB *and *HPRT1 *as the most stable genes in the *in vivo *embryos and *UBC*, *RPL32*, *GAPDH *and *ACTB *in both *in vitro *populations. When *in vivo *and *in vitro *embryos were combined, *UBC*, *ACTB*, *RPL32 *and *GAPDH *were found to be the most stable. *SDHA *and *H2A/I *appeared to be highly regulated.

**Conclusions:**

Based on these results, the geometric mean of *UBC*, *ACTB*, *RPL32 *and *GAPDH *is to be recommended for accurate normalization of quantitative real-time PCR data in equine *in vivo *and *in vitro *produced blastocysts.

## Background

Conventional IVF has been rather unsuccessful in horses. To overcome this barrier ICSI has been introduced and has resulted in transferable blastocysts that were used for research and commercial purposes [1-3]. The ability of *in vitro *produced blastocysts to establish normal pregnancies is comparable to that of the *in vivo *derived ones and, contrary to other species, *in vitro *produced equine blastocysts tolerate freezing better than *in vivo *produced ones and they are able to establish pregnancies after thawing [1]. Despite these successes, there is a great variability in oocyte quality and culture conditions resulting in a low percentage of blastocyst formation *in vitro *with great variations amongst laboratories. Compared to their *in vivo *counterparts, *in vitro *blastocysts are retarded in the kinetics of development, are smaller with fewer cells, show more apoptosis and higher levels of chromosomal abnormalities [4-6]. Moreover intra-uterine transfer of *in vitro *produced embryos can give rise to abnormal pregnancies with the development of a trophoblastic vesicle without an embryo proper [2]. Understanding the fundamental difference between equine *in vivo *versus *in vitro *embryos may prove beneficial in the development of equine assisted reproductive techniques.

RT-qPCR is a highly specific and sensitive tool to compare mRNA expression levels of specific genes [7]. Not only the RNA quality, the RT, the reagents and the protocol are critical factors, the analytical method can also influence the results dramatically [7,8]. All data must be normalized for technical differences between samples. The method of preference consists in normalization to internal reference genes, which should be constitutively expressed without influence of the experimental treatment. Since there is no universal reference gene with a constant expression in all tissues, optimal reference genes need to be selected for each system [9]. The use of a single reference gene or of multiple unstable reference genes may lead to erroneous normalization [10]. Therefore the geometric mean of carefully selected genes is recommended for reliable normalization [11].

Reference genes have been validated in preimplantation embryos of cattle [12], mice [13], pigs [14] and rabbits [15]. However gene stability in mammalian embryos turned out to be species specific, which implies the need of reference gene selection for the study of equine embryos. Gene expression studies in equine species are relatively scarce. Real-time PCR has been conducted on equine conceptuses to evaluate expression of progesterone and oestrogen receptors and on equine blastocysts to identify POU5F1 expression, but in these studies only one reference gene, β-actin, was used [16,17]. While sets of reference genes have been identified for equine skin [18] and lymphocytes [19], the most reliable reference genes in equine embryos have not been studied before.

The aim of this study was to identify a set of stable reference genes for equine *in vivo *derived embryos and fresh and frozen-thawed *in vitro *embryos.

## Methods

The *in vivo *blastocyst collection procedures used were approved by the ethics committee of the faculty of veterinary medicine (reference number EC 2007/009). Cycling mares were followed up by transrectal ultrasound. When the follicle size exceeded 35 mm in the presence of an edematous uterus, 3000 IU hCG (Chorulon, Intervet, Belgium) was injected intravenously. The next day the mare was inseminated with fresh semen. Ultrasound was performed daily until ovulation was detected. If the mare did not ovulate within 48 hours after AI, insemination was repeated. The mares' uterus was flushed 7 days after ovulation and recovered embryos were washed in DPBS (14190, Gibco, Invitrogen, Belgium) and conserved individually at -80°C in lysis buffer (10% RNasin Plus RNase inhibitor (Promega, The Netherlands), 5% dithiothreitol (Promega, The Netherlands), 0.8% Igepal CA-630 (Sigma, Belgium) in RNase free water until further analysis.

For the production of fresh *in vitro *blastocysts, ovaries were recovered from slaughtered mares and follicles ≥5 mm were aspirated with a vacuum pump at a negative pressure of 100 mm Hg. The follicle wall was scraped with the aspirating 16 gauge needle and flushed with 0.5% (v/v) heparin (H 1027, Sigma, Belgium) in DPBS (14190, Gibco, Invitrogen, Belgium). Oocytes were matured for 28 h in DMEM-F12 based medium [1] at 38.5°C in 5% CO_2 _in air. The MII oocytes were fertilized by conventional ICSI as described by Tremoleda et al. [20]. Frozen-thawed sperm was used from the same stallion that was used for production of *in vivo *embryos. *In vitro *culture was performed for 8.5 to 9.5 days in DMEM-F12 (D 2906, Sigma, Belgium) with 10% FCS (F 9665, Sigma, Belgium) or 5% FCS and 5% SR (10828-028, Gibco, Invitrogen, Belgium) at 38.5°C in 5% CO_2_, 5% O_2 _and 90% N_2_. Blastocysts were stored individually at -80°C in lysis buffer until further analysis.

The frozen-thawed *in vitro *embryos were produced in Italy. Briefly, oocytes were recovered from abbatoir ovaries by scraping and washing all the follicles between 5 and 30 mm. Oocytes were washed in HEPES buffered TCM199 and transferred for 24 h into DMEM-F12 based maturation medium as previously described [1]. After removal of all cumulus cells, the oocytes with a polar body were fertilized by ICSI with frozen-thawed sperm from stallions of proven fertility. Injected oocytes were cultured up to day 8 in 20 μl drops of modified SOF (from day 6 half SOF: half DMEM-F12) at 38.5°C in 5% CO_2_, 5% O_2 _and 90% N_2_. Embryos that reached the blastocyst stage were loaded individually into 0.25 ml straws, frozen with a slow cooling protocol (0.5°C/min up to -32°C) in glycerol and stored in liquid N_2_. The embryos were shipped to Belgium in dry ice and upon arrival they were immediately transferred into lysis buffer with a minimum of medium and conserved at -80°C until further analysis.

In all groups only blastocysts which were not hatched and had good morphological characteristics were retained.

For each of the 3 groups 8 embryos were analysed separately. Total RNA was extracted from single embryos with the PicoPure RNA-isolation Kit (Arcturus, USA), treated with RQ1 DNAse (Promega, The Netherlands) and purified over a spin column (Microcon YM-100, Millipore, Belgium). After minus RT control with primers for *GAPDH *to check for contaminating genomic DNA, RNA was concentrated by precipitation with 3 M sodium acetate and ethanol. RNA amplification and conversion into cDNA was performed by means of the WT-Ovation RNA Amplification System (NuGEN, USA) according to the manufacturers' instructions and the cDNA was purified again over a spin column. Amplified cDNA samples were diluted 10-20 times, depending on the yield, in 10 mM Tris HCL pH 8.0 and stored at -80°C.

Eight reference genes were selected based on previous studies [12,18,19]. The selected genes (*ACTB*, *GAPDH*, *H2A/I*, *HPRT*, *RPL32*, *SDHA*, *TUBA4A *and *UBC*) belong to different functional classes, which reduces the chance of co-regulation.

Primers for *ACTB*, *HPRT1*, *RPL32*, *TUBA4A *and *UBC *were provided by Bogaert and colleagues [18]. The other primers were designed by means of Primer3 software http://frodo.wi.mit.edu/primer3/, based on horse sequences found in the NCBI GenBank http://www.ncbi.nlm.nih.gov/. Primers were selected over intron-exon boundaries, tested using a BLAST analysis against the NCBI database and verified using MFold http://frontend.bioinfo.rpi.edu/applications/mfold/cgi-bin/dna-form1.cgi. The optimal primer annealing temperatures were determined on cDNA of equine mixed tissues. A melt curve analysis followed by agarose gel electrophoresis was performed to test for primer-dimer formation and specificity of the amplicons. All primers are listed in Table 1.

**Table 1:** Primers

Gene	GenBank accession number	Sequence	Amplicon size (bp)	Ta (°C)	RTprimerDB ID	C_q _range
ACTB	AF035774	CCAGCACGATGAAGATCAAG GTGGACAATGAGGCCAGAAT	88	60	7848	16.3-23.8
GAPDH	XM_001496020.1	CAGAACATCATCCCTGCTTC ATGCCTGCTTCACCACCTTC	187	59	7849	14.2-24.5
H2A/I	XM_001497311.2	ATATTCAGGCCGTGCTGCT TTTGGGTTTCAAAGCGTTTC	105	60	*	23.5-37.3
HPRT1	AY372182	GGCAAAACAATGCAAACCTT CAAGGGCATATCCTACGACAA	163	57	7850	17.6-25.8
RPL32	XM_001492042.2	AGCCATCTACTCGGCGTCA TCCAATGCCTCTGGGTTTC	149	60	*	16.8-25.9
SDHA	XM_001490889	TCCATCGCATAAGAGCAAAG GGTGGAACTGAACGAACTCC	159	59	7851	20.7-33.3
TUBA4A	XM_001491910.2	GCCCTACAACTCCATCCTGA ATGGCTTCATTGTCCACCA	78	60	*	16.6-27.9
UBC	AF506969	GCAAGACCATCACCCTGGA CTAACAGCCACCCCTGAGAC	206	60	7874	15.5-23.7

For each reference gene, the NCBI GenBank accession number, the sequence of both forward and reverse primer, the size of the amplicon and the optimal primer annealing temperature are listed. The RTPrimerDB ID http://rtprimerdb.org/ is also reported, except for the primers for which there is not yet an official reference sequence available in the database (*).

All reactions were executed in duplicate for 8 embryos in each of the three groups and a blank was included in each run.

The diluted cDNA (2.5 μl), 0.33 μM of both forward and reverse primers and 4 μl of RNAse free water were added to 7.5 μl of KAPA SYBR FAST qPCR Master Mix (KAPA Biosystems) to a final volume of 15 μl. The reactions were performed on the iCycler iQ Real-Time PCR Detection System (Bio-Rad, Belgium).

The PCR program started with an initial denaturation at 95°C for 3 minutes to activate the DNA polymerase. Then 45 cycles were performed with a denaturation step at 95°C for 20 seconds followed by an annealing/extension step at the primer specific annealing temperature for 40 seconds during which fluorescence was measured. A dilution series with pooled cDNA from all 24 embryos was included for each gene to acquire PCR efficiencies based on a relative standard curve. Calculation of the C_q _values, PCR efficiencies, correlation coefficients and analysis of the melting curves was performed by means of iCycler iQ Optical System Software Version 3.0a. The standard errors of the PCR efficiencies were obtained by the qBasePlus software http://www.qbaseplus.com/.

The expression stabilities were evaluated using the geNorm software for Microsoft Excel [11]. This program ranks the genes based on the internal control gene stability parameter M. Stepwise exclusion of the gene with the highest M value and recalculation results in a ranking of the reference genes. Lower M values represent higher expression stabilities. Furthermore the minimum number of genes required for the calculation of a reliable normalization factor is determined.

## Results and discussion

For the recovery of the *in vivo *embryos (n = 8) 13 uterine flushes were performed in a population of 8 mares (recovery rate: 61.5%). To collect the fresh *in vitro *embryos (n = 8) 123 ovaries were recovered in 5 experiments which gave rise to a total number of 365 oocytes. Maturation was completed and ICSI was performed in 209 oocytes (57%) and 74% of these injected oocytes cleaved. Of the cleaved oocytes 5.8% reached the blastocyst stage. The 8 *in vitro *blastocysts which were used for genetic analysis were produced in 2 of those 5 experiments where the blastocyst percentage was 7.3%.

Frozen embryos were produced in experiments for quality control purposes. They were frozen on day 8 at the early blastocyst or blastocyst stage.

Prior to the start of the experiment, PCR was tested on one of the frozen-thawed embryos to evaluate possible effects of remnants of the cryoprotectant. Both undiluted and 25× diluted cDNA resulted in clear electrophoretic bands of expected size for *UBC *and *ACTB*. Only a minimal amount of cryoprotectant accompanying the embryo was transferred to and diluted in the lysis buffer. Importantly C_q_-values of the fresh and frozen-thawed embryos remained in the same range.

Because only a small quantity of RNA can be extracted from one embryo, an RNA pre-amplification step was included in the protocol. Ribo-SPIA, the isothermal messenger RNA amplification method used, has been shown to be accurate and reproducible [21]. High sensitivity permits quantification of low abundance transcripts and there is a good correlation in differential gene expression between amplified and nonamplified cDNA [21].

Dilution series of all candidate reference genes gave PCR-efficiencies between 97.1% and 100% and linear correlation coefficients that varied from 0.973 to 1.000. In table 2 the efficiencies and their standard errors are listed.

**Table 2:** PCR efficiency and the respectively standard error

Gene	Efficiency (%)	Standard error
ACTB	98.1	0.01
GAPDH	100	0.021
H2A/I	100	0.067
HPRT1	100	0.037
RPL32	100	0.071
SDHA	99.3	0.033
TUBA4A	100	0.017
UBC	97.1	0.017

One *in vivo *and one fresh *in vitro *embryo consistently resulted in C_q_-values around 40. Probably the amplification went wrong and both samples were deleted from further analysis.

The selection of appropriate reference genes can be achieved by evaluating RT-qPCR data with statistical algorithms. The uniformity in gene ranking between geNorm [11], NormFinder [22] and BestKeeper [23] has been shown to be high [19,24].

**Figure 1:**
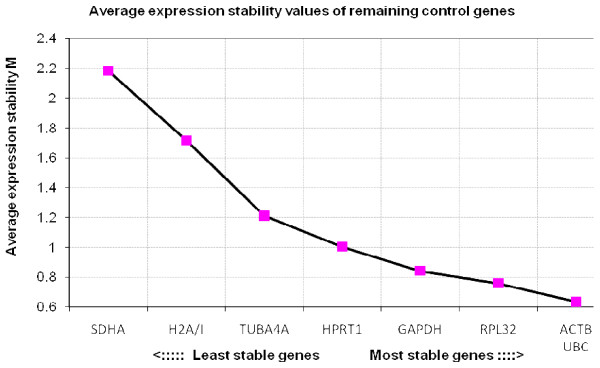
**Average expression stability values of equine *in vivo *and *in vitro *embryos**. The average stability values of the control genes were calculated with geNorm. When *in vivo *embryos and fresh and frozen-thawed *in vitro *embryos were combined, *UBC*, *ACTB*, *RPL32 *and *GAPDH *were found to be the most stable.

**Figure 2:**
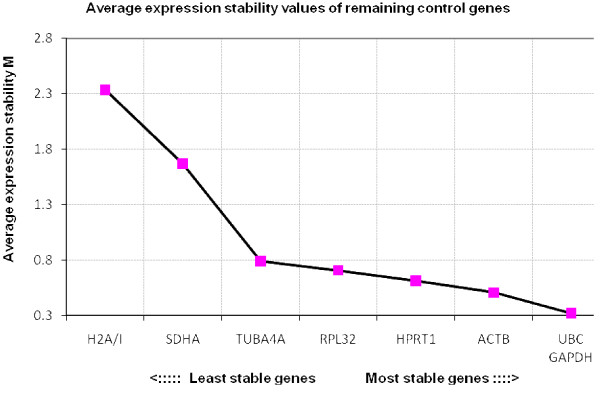
**Average expression stability values of equine *in vivo *embryos**. The average stability values of the control genes were calculated with geNorm. In the population of the equine *in vivo *embryos *UBC*, *GAPDH*, *ACTB *and *HPRT1 *were found to be the most stable.

**Figure 3:**
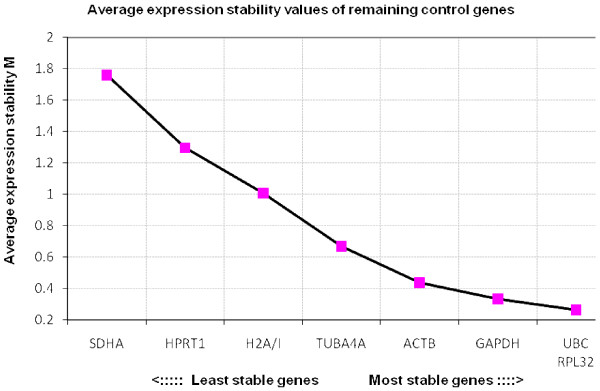
**Average expression stability values of fresh equine *in vitro *embryos**. The average stability values of the control genes were calculated with geNorm. In the population of the fresh equine *in vivo *embryos *UBC*, *RPL32*, *GAPDH *and *ACTB *were found to be the most stable.

**Figure 4:**
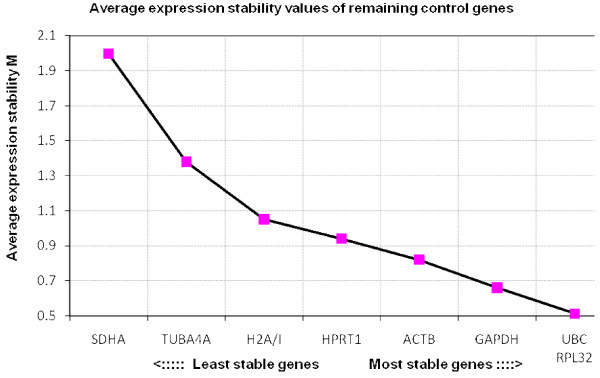
**Average expression stability values of frozen-thawed equine *in vitro *embryos**. The average stability values of the control genes were calculated with geNorm. In the population of the fresh equine *in vivo *embryos *UBC*, *RPL32*, *GAPDH *and *ACTB *were found to be the most stable.

Results of the reference gene stability, as determined by geNorm, are shown in Figure 1, 2, 3 and 4. When all three groups of embryos were included, the most stable genes were *UBC*, *ACTB*, *RPL32 *and *GAPDH*. The M values of these genes range between 0.6 and 0.9, which indicates relatively good stability.

The order of the 4 most stable genes, *UBC*, *RPL32*, *GAPDH *and *ACTB*, is identical in both *in vitro *groups. This is an interesting finding as there was a difference in embryo culture conditions and as the cryopreservation could have affected the results. Three of those 4 genes, *UBC*, *GAPDH *and *ACTB*, represent the most stable genes in the *in vivo *embryos. In murine embryos the order of stability of reference genes has also been found to be similar in both *in vitro *and *in vivo *embryos [13]. In the latter study however, the stability measure values M were described to be higher in the *in vitro *samples compared to the *in vivo *samples, which was not the case in the equine embryos. *SDHA *and *H2A/I *generally turned out to be highly regulated.

These results differ from those in embryos from other species and from those in other equine tissues. *SDHA*, which was highly regulated in equine embryos, appeared to be very stable in bovine embryos [12] and equine lymphocytes [19]. This is also the case for *H2A/I*, which was preferred as a reference gene in embryos of mice [13] and rabbits [15]. *ACTB *on the other hand appears fairly stable in equine embryos, although it is highly regulated in bovine embryos [12]. This again demonstrates the necessity to validate the genes according to the species and the tissue type.

**Figure 5:**
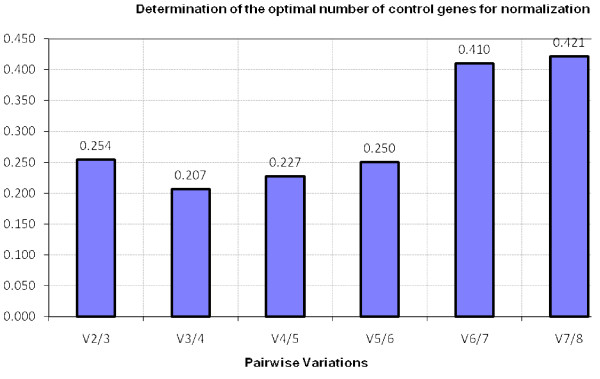
**Determination of the optimal number of control genes for normalization**. The optimal number of control genes for normalization was calculated by geNorm. The value of the pairwise variations reduces until 0.207 for V_3/4_, which indicates that the inclusion a fourth reference gene contributes to the stability. Therefore the average of the 4 most stable genes is recommended to determine a reliable NF.

The use of the geometric mean of several internal control genes as a normalization factor (NF) is more accurate than the use of a single reference gene. Inclusion of unstable genes on the other hand negatively influences the NF and the number of reference genes used to calculate this NF is a trade-off between practical considerations and accuracy. The optimal number of genes was determined with geNorm by means of the pairwise variations (V_n/n+1_) between the sequential normalization factors (NF_n _and NF_n+1_) after successive inclusion of less stable reference genes (Figure 5). The value of the pairwise variations reduces until 0.207 for V_3/4_. This suggests that the inclusion of a fourth reference gene contributes to the stability. Therefore it is recommended to use the 4 most stable genes, *UBC*, *ACTB*, *RPL32 *and *GAPDH*. The high values of V_6/7 _and V_7/8 _represent the instability of *H2A/I *and *SDHA*, which is also clear in the steep rise in Figure 1.

In conclusion, the geometric mean of *ACTB*, *UBC*, *RPL32 *and *GAPDH *is recommended for normalization of RT-qPCR data in experiments with equine *in vivo *and *in vitro *blastocysts.

## Abbreviations

ACTB: beta actin; GAPDH: glyceraldehyde-3-phosphate dehydrogenase; H2A/I: histone H2A type 1-C; HPRT: hypoxanthine phosphoribosyltransferase 1; RPL32: ribosomal protein L32; SDHA: succinate dehydrogenase complex; subunit A; TUBA4A: tubulin; alpha 4a; UBC: ubiquitin C; IVF: *in vitro *fertilization; ICSI: intracytoplasmic sperm injection; RT-qPCR: reverse transcription quantitative real-time polymerase chain reaction; RT: reverse transcription; hCG: human chorionic gonadotropin. AI: artificial insemination; DPBS: Dulbecco's Phosphate Buffered Saline; DMEM-F12: Dulbecco's Modified Eagle Medium: Nutrient Mixture F-12; MII: metaphase II; FCS: fetal calf serum; SR: serum replacement; HEPES: 4-(2-hydroxyethyl)-1-piperazineethanesulfonic acid; TCM199: Tissue Culture Medium199; SOF: synthetic oviduct fluid; MEM: Eagle's minimal essential medium; FAF-BSA: fatty acid-free bovine serum albumin; C_q_value: quantification cycle value.

## Competing interests

The authors declare that they have no competing interests.

## Authors' contributions

KS performed all experiments and data analysis and was the primary author of the manuscript. KG provided support in all genetical procedures and in data analysis. JG, MH and EVH helped with the collection of the *in *vivo embryos. SC and CG provided the frozen-thawed *in vitro *embryos and helped with the optimization of the protocol for the fresh *in vitro *embryos. JV supported the data analysis. AVS and LP participated in the design of the project and supervised the study. All authors read and approved the final manuscript.
